# [Retracted] Placenta-derived mesenchymal stem cells improve airway hyperresponsiveness and inflammation in asthmatic rats by modulating the Th17/Treg balance

**DOI:** 10.3892/mmr.2025.13601

**Published:** 2025-06-23

**Authors:** Yingying Li, Hongbo Li, Yinyin Cao, Fuling Wu, Wenbin Ma, Yuesi Wang, Shuzhen Sun

Mol Med Rep 16: 8137–8145, 2017; DOI: 10.3892/mmr.2017.7605

Following the publication of the above paper, it was drawn to the Editor's attention by a concerned reader that, regarding the flow cytometric plots shown in Fig. 4A and B, and Fig. 4D and E, respectively on p. 8142, these two pairs of plots appeared to show similar groupings of dots, which would not have been anticipated if these experiments had been performed discretely under different experimental conditions, suggesting a fundamental flaw either in the way in which these experiments were performed or in how the results were outputted.

The Editor of *Molecular Medicine Reports* has decided that this paper should be retracted from the Journal on account of a lack of confidence in the presented data. The authors were asked for an explanation to account for these concerns, but the Editorial Office did not receive a satisfactory reply. The Editor apologizes to the readership for any inconvenience caused.

